# Supplementary material for: Adverse Events in 1406 Patients Receiving 13,780 Cycles of Azacitidine within the Austrian Registry of Hypomethylating Agents—A Prospective Cohort Study of the AGMT Study-Group
Source: Cancers (Basel). 2022 May 17;14(10):2459. doi: 10.3390/cancers14102459 (PMC9140081; doi:10.3390/cancers14102459)
Supplement: Supplementary file 1 [file cancers-14-02459-s001.zip › cancers-1688279-supplementary.pdf]

Supplemental Table S1.·CTCAE v5.0 criteria used for the calculation of treatment emergent adverse events (TEAEs) [26]**.**

MedDRA Code	MedDRA SOC	CTCAE Term	Grade 1	Grade 2	Grade 3	Grade 4	Grade 5
10002272	Blood and lymphatic system disorders	Anemia	Hemoglobin (Hgb) <LLN - 10.0 g/dL; <LLN - 6.2 mmol/L; <LLN - 100 g/L	Hgb <10.0 - 8.0 g/dL; <6.2 - 4.9 mmol/L; <100 - 80g/L	Hgb <8.0 g/dL; <4.9 mmol/L; <80 g/L; transfusion indicated	Life-threatening consequences; urgent intervention indicated	Death
10024378	Blood and lymphatic system disorders	Leukocytosis	-	-	>100,000/mm3	Clinical manifestations of leucostasis; urgent intervention indicated	Death
10001551	Investigations	Alanine aminotransferase increased	>ULN - 3.0 x ULN if baseline was normal; 1.5 - 3.0 x baseline if baseline was abnormal	>3.0 - 5.0 x ULN if baseline was normal; >3.0 - 5.0 x baseline if baseline was abnormal	>5.0 - 20.0 x ULN if baseline was normal; >5.0 - 20.0 x baseline if baseline was abnormal	>20.0 x ULN if baseline was normal; >20.0 x baseline if baseline was abnormal	-
10003481	Investigations	Aspartate aminotransferase increased	>ULN - 3.0 x ULN if baseline was normal; 1.5 - 3.0 x baseline if baseline was abnormal	>3.0 - 5.0 x ULN if baseline was normal; >3.0 - 5.0 x baseline if baseline was abnormal	>5.0 - 20.0 x ULN if baseline was normal; >5.0 - 20.0 x baseline if baseline was abnormal	>20.0 x ULN if baseline was normal; >20.0 x baseline if baseline was abnormal	-
10005364	Investigations	Blood bilirubin increased	>ULN - 1.5 x ULN if baseline was normal; > 1.0 - 1.5 x baseline if baseline was abnormal	>1.5 - 3.0 x ULN if baseline was normal; >1.5 - 3.0 x baseline if baseline was abnormal	>3.0 - 10.0 x ULN if baseline was normal; >3.0 - 10.0 x baseline if baseline was abnormal	>10.0 x ULN if baseline was normal; >10.0 x baseline if baseline was abnormal	-
10011368	Investigations	Creatinine increased	>ULN - 1.5 x ULN	>1.5 - 3.0 x baseline; >1.5 - 3.0 x ULN	>3.0 x baseline; >3.0 - 6.0 x ULN	>6.0 x ULN	-
10025256	Investigations	Lymphocyte count decreased	<LLN - 800/mm3; <LLN - 0.8 x 10e9/L	<800 - 500/mm3; <0.8 - 0.5 x 10e9 /L	<500 - 200/mm3; <0.5 - 0.2 x 10e9 /L	<200/mm3; <0.2 x 10e9 /L	-
10029366	Investigations	Neutrophil count decreased	<LLN - 1500/mm3; <LLN - 1.5 x 10e9 /L	<1500 - 1000/mm3; <1.5 - 1.0 x 10e9 /L	<1000 - 500/mm3; <1.0 - 0.5 x 10e9 /L	<500/mm3; <0.5 x 10e9 /L	-
10035528	Investigations	Platelet count decreased	<LLN - 75,000/mm3; <LLN - 75.0 x 10e9 /L	<75,000 - 50,000/mm3; <75.0 - 50.0 x 10e9 /L	<50,000 - 25,000/mm3; <50.0 - 25.0 x 10e9 /L	<25,000/mm3; <25.0 x 10e9 /L	-
10049182	Investigations	White blood cell decreased	<LLN - 3000/mm3; <LLN - 3.0 x 10e9 /L	<3000 - 2000/mm3; <3.0 - 2.0 x 10e9 /L	<2000 - 1000/mm3; <2.0 - 1.0 x 10e9 /L	<1000/mm3; <1.0 x 10e9 /L	-

MedDRA SOC indicates Medical Dictionary for Regulatory Archives System Organ Classes.

Supplemental Table S2. Comorbidities at azacitidine treatment start of patients in the Austrian Registry of Hypomethylating Agents.

Comorbidities, n (%)	Total cohort (N=1406)	MDS (N=504)	CMML (N=133)	AML (N=769)
Thromboembolic	119 (8.5)	35 (6.9)	9 (6.8)	75 (9.8)
Renal	238 (16.9)	76 (15.1)	36 (27.1)	126 (16.4)
Hepatic	140 (10.0)	47 (9.3)	17 (12.8)	76 (9.9)
Diabetes	259 (18.4)	93 (18.5)	25 (18.8)	141 (18.3)
Solid tumor	240 (17.1)	90 (17.9)	24 (18.0)	126 (16.4)
Pulmonary	196 (13.9)	69 (13.7)	17 (12.8)	110 (14.3)
Cardiac	528 (37.6)	175 (34.7)	60 (45.1)	293 (38.1)
Obesity (BMI > 35 kg/m^2)	113 (8.0)	38 (7.5)	7 (5.3)	68 (8.8)
Cerebrovascular disease	91 (6.5)	26 (5.2)	10 (7.5)	55 (7.2)
Rheumatologic	58 (4.1)	21 (4.2)	5 (3.8)	32 (4.2)
Peptic ulcer	32 (2.3)	11 (2.2)	4 (3.0)	17 (2.2)
Inflammatory bowel disease	15 (1.1)	5 (1.0)	3 (2.3)	7 (0.9)
Psychiatric	101 (7.2)	25 (5.0)	7 (5.3)	69 (9.0)

Supplemental Table S3. *T*reatment characteristics and outcomes of patients treated with azacitidine within the Austrian Registry of Hypomethylating Agents.

	Total cohort (N=1406)	MDS (N=504)	CMML (N=133)	AML (N=769)	p-value
Treatment duration, months: Mean (SD)	9.8 (13.57)	12.0 (15.5)	13.1 (15.68)	7.7 (11.31)	<0.0001
Median (IQR)	5.1 (1.9-12.1)	6.6 (3.0-14.5)	7.6 (3.4-17.2)	3.9 (1.2-9.7)	
Min-Max	0.0-102.2	0.1-102.2	0.1-95.3	0.0-101.1	
Number of azacitidine cyles, n: Mean (SD)	9.7 (12.35)	11.9 (14.63)	12.9 (14.86)	7.7 (9.62)	<0.0001
Median (IQR)	5 (2.0-12.0)	6.5 (3.5-14.5)	8.0 (4.0-16.0)	4.0 (2.0-10.0)	
Min-Max	1.0-111.0	1.0-111.0	1.0-100.0	1.0-75.0	
Overall survival, months: Mean (SD)	14.1 (15.84)	19.1 (20.07)	17.2 (14.53)	10.7 (11.92)	<0.0001
Median (IQR)	9.7 (3.8-18.8)	13.8 (6.9-23.7)	14.1 (6.9-24.0)	7.3 (3.1-14.4)	
Min-Max	0.0-161.6	0.4-161.6	0.4-80.7	0.0-108.4	
Disease-free survival, months: Mean (SD)	5.2 (11.08)	5.9 (12.49)	8.7 (14.01)	4.2 (9.24)	<0.0001
Median (IQR)	1.6 (0.9-4.8)	1.9 (0.9-5.2)	3.4 (1.2-9.7)	1.2 (0.9-3.7)	
Min-Max	0.0-161.6	0.0-161.6	0.0-95.3	0.0-108.1	
Follow-up, months: Mean (SD)	16.8 (19.9)	22.7 (23.82)	18.6 (16.32)	12.6 (16.31)	<0.0001
Median (IQR)	10.7 (4.1-21.2)	15.4 (7.6-29.0)	14.5 (7.0-24.7)	7.9 (3.2-16.0)	
Min-Max	0.0-161.6	0.3-161.6	0.2-95.3	0.0-134.0	
Azacitidine treatment line: 1st, n (%)	838 (59.6)	397 (78.8)	95 (71.4)	346 (45.0)	<0.0001
2nd	301 (21.4)	82 (16.3)	29 (21.8)	190 (24.7)	
3rd	128 (9.1)	16 (3.2)	8 (6.0)	104 (13.5)	
4th	76 (5.4)	7 (1.4)	1 (0.8)	68 (8.8)	
>5th	63 (4.5)	2 (0.4)	0 (0.0)	61 (7.9)	
Best response:[1] Complete remission, n (%)	154 (11.0)	56 (11.1)	22 (16.5)	76 (9.9)	<0.0001
Complete remission with incomplete recovery	54 (3.8)	29 (5.8)	9 (6.8)	16 (2.1)	
Morphologic leukemia free state	84 (6.0)	44 (8.7)	9 (6.8)	31 (4.0)	
Partial remission	25 (1.8)	3 (0.6)	2 (1.5)	20 (2.6)	
Hematologic improvement	322 (22.9)	142 (28.2)	38 (28.6)	142 (18.5)	
Progressive disease	48 (3.4)	20 (4.0)	2 (1.5)	26 (3.4)	
No response	719 (51.1)	210 (41.7)	51 (38.3)	458 (59.6)	
Death within 30 days: Yes, n (%)	78 (5.5)	13 (2.6)	4 (3.0)	61 (7.9)	<0.0001
No	1328 (94.5)	491 (97.4)	129 (97.0)	708 (92.1)	
Alive at 1 year: Yes, n (%)	692 (49.2)	314 (62.3)	83 (62.4)	295 (38.4)	<0.0001
No	714 (50.8)	190 (37.7)	50 (37.6)	474 (61.6)	
Alive at 3 years: Yes, n (%)	251 (17.9)	141 (28.0)	27 (20.3)	83 (10.8)	<0.0001
No	1155 (82.1)	363 (72.0)	106 (79.7)	686 (89.2)	

[1]According to European Leukemia Net (ELN) criteria [27].

Supplemental Table S4. Infectious complications documented in the Austrian Registry of Hypomethylating Agents.

	Total cohort (N=1406)	MDS (N=504)	CMML (N=133)	AML (N=769)	p-value
Catheter related infection Grade1-2, n (%)	13 (0.9)	4 (0.8)	0 (0.0)	9 (1.2)	0.9244
Grade 3-4	6 (0.4)	2 (0.4)	2 (1.5)	2 (0.3)	
Herpes simplex reactivation Grade1-2, n (%)	44 (3.1)	15 (3.0)	4 (3.0)	25 (3.3)	0.9769
Grade 3-4	2 (0.1)	2 (0.4)	0 (0.0)	0 (0.0)	
Pneumonia Grade1-2, n (%)	207 (14.7)	79 (15.7)	15 (11.3)	113 (14.7)	0.4446
Grade 3-4	80 (5.7)	24 (4.8)	4 (3.0)	52 (6.8)	
Sepsis Grade1-2, n (%)	14 (1.0)	4 (0.8)	1 (0.8)	9 (1.2)	0.2252
Grade 3-4	37 (2.6)	12 (2.4)	1 (0.8)	24 (3.1)	
Shingles Grade1-2, n (%)	18 (1.3)	4 (0.8)	3 (2.3)	11 (1.4)	0.3529
Grade 3-4	0 (0.0)	0 (0.0)	0 (0.0)	0 (0.0)	
Upper resp. tract infection Grade1-2, n (%)	227 (16.1)	93 (18.5)	22 (16.5)	112 (14.6)	0.1811
Grade 3-4	13 (0.9)	3 (0.6)	1 (0.8)	9 (1.2)	
Urinary tract infection Grade1-2, n (%)	106 (7.5)	40 (7.9)	11 (8.3)	55 (7.2)	0.8263
Grade 3-4	12 (0.9)	6 (1.2)	0 (0.0)	6 (0.8)	

Supplemental Table S5. Baseline characteristics and adverse events of the decitabine cohort

	Total cohort (N=67)
Baseline characteristics	
Diagnosis at decitabine start: MDS, n (%)	29 (43.2)
CMML	8 (11.9)
AML	29 (43.2)
Unknown	1 (1.4)
Mean age, years	72.5
Median (IQR)	75 (69-79)
Min-max	25-86
≥ 75 years, n (%)	31 (31.9)
Unknown	0 (0.0)
Sex: Female, n (%)	16 (23.8)
Male	51 (76.2)
Unknown	0 (0.0)
ECOG-PS: 0-1, n (%)	6 (6.1)
2-4	0 (0.0)
Unknown	61 (91.0)
HCT-CI risk group: Low risk, n (%)	26 (38.8)
Intermediate risk	23 (34.3)
High risk	18 (26.8)
Unknown	0 (0.0)
Treatment-related disease: No, n (%)	56 (83.5)
Yes	10 (14.9)
Unknown	1 (1.4)
IPSS cytogenetic risk: Good, n (%)	12 (12.3)
Intermediate	7 (10.4)
Poor	5 (7.4)
Not evaluable	4 (5.9)
Unknown	39 (58.2)
R-IPSS cytogenetic risk: Very good, n (%)	0 (0.0)
Good	12 (17.9)
Intermediate	9 (13.4)
Poor	2 (2.9)
Very poor	1 (1.4)
Not evaluable	4 (5.9)
Unknown	39 (58.2)
IPSS risk group: lower-risk2, n (%)	4 (5.9)
Higher-risk3	18 (26.8)
Unknown	45 (67.1)
Red blood cell transfusion dependence: Yes, n (%)	2 (2.9)
No	17 (25.3)
Unknown	48 (71.6)
Platelet transfusion dependence: Yes, n (%)	6 (8.9)
No	13 (19.4)
Unknown	48 (71.6)
Calculated adverse events	
Neutropenia:1 Grade1-2, n (%)	9 (13.4)
Grade 3-4	18 (26.8)
Lymphopenia:1 Grade 1-2, n (%)	9 (13.4)
Grade 3-4	5 (7.4)
Anemia:1 Grade 1-2, n (%)	9 (13.4)
Grade 3-4	20 (29.8)
Thrombopenia:1 Grade 1-2, n (%)	6 (8.9)
Grade 3-4	17 (25.3)
Bilirubin increase:2 Grade1-2, n (%)	5 (7.4)
Grade 3-4	8 (11.9)

GOT increase:2 Grade 1-2, n (%)	8 (11.9)
Grade 3-4	0 (0.0)
GPT increase:2 Grade 1-2, n (%)	10 (14.9)
Grade 3-4	0 (0.0)
Creatinine increase:2 Grade 1-2, n (%)	8 (11.9)
Grade 3-4	0 (0.0)
Documented adverse events	
Pyrexia: Grade 1-2, n (%)	0 (0.0)
Grade 3-4	6 (8.9)
Febrile neutropenia: Grade 1-2, n (%)	0 (0.0)
Grade 3-4	9 (13.4)
Pneumonia Grade 1-2, n (%)	1 (1.4)
Grade 3-4	7 (10.4)
Upper resp. infection: Grade 1-2, n (%)	1 (1.4)
Grade 3-4	6 (8.9)
Nausea: Grade 1-2, n (%)	1 (1.4)
Grade 3-4	1 (1.4)
Diarrhea: Grade 1-2, n (%)	0 (0.0)
Grade 3-4	3 (4.4)
Constipation: Grade 1-2, n (%)	0 (0.0)
Grade 3-4	0 (0.0)
Urinary tract infection: Grade 1-2, n (%)	0 (0.0)
Grade 3-4	0 (0.0)
Skin/mucosal infection: Grade 1-2, n (%)	0 (0.0)
Grade 3-4	3 (4.4)
Bacterial infection other: Grade 1-2, n (%)	1 (1.4)
Grade 3-4	2 (2.9)
Injection site reaction: Grade 1-2, n (%)	0 (0.0)
Grade 3-4	0 (0.0)
Fatigue: Grade 1-2, n (%)	3 (4.4)
Grade 3-4	2 (2.9)
Pain: Grade 1-2, n (%)	4 (5.9)
Grade 3-4	5 (7.4)
